# Re-Analysis of 16S Amplicon Sequencing Data Reveals Soil Microbial Population Shifts in Rice Fields under Drought Condition

**DOI:** 10.1186/s12284-020-00403-6

**Published:** 2020-07-02

**Authors:** Seok-Won Jang, Myeong-Hyun Yoou, Woo-Jong Hong, Yeon-Ju Kim, Eun-Jin Lee, Ki-Hong Jung

**Affiliations:** 1 Division of Life Sciences, College of Life Sciences and Biotechnology, Korea University, Seoul, 02841 South Korea; 2 Graduate School of Biotechnology, Kyung Hee University, Yongin, 17104 South Korea; 3 Graduate School of Biotechnology & Crop Biotech Institute, Kyung Hee University, Yongin, 17104 South Korea; 4 Department of Oriental Medicine Biotechnology, College of Life Sciences, Kyung Hee University, Yongin, 17104 South Korea

**Keywords:** Meta-analysis, Rice, Drought, Microbiome, Amplicon sequencing

## Abstract

Rice (*Oryza sativa. L*) has been intensively studied to ensure a stable global supply of this commodity in the face of rapid global climate change. A critical factor that decreases crop yield is drought, which has been analyzed in various ways through many researches. Microbiome-based studies of rice investigate the symbiosis between rice and bacteria, which has been proposed as a way to overcome problems caused by drought. Several rice-associated metagenomic profiles obtained under drought conditions have been reported since the advent of next generation sequencing (NGS) technology. To elucidate the future diversity of plants and microorganisms and to promote sustainable agriculture, we reanalyzed 64 of the publicly available 16S amplicon sequencing data produced under drought condition. In the process of integrating data sets, however, we found an inconsistency that serves as a bottleneck for microbiome-based sustainability research. While this report provides clues about the composition of the microbiome under the drought conditions, the results are affected by differences in the location of the experiments, sampling conditions, and analysis protocols. Re-analysis of amplicon sequencing data of the soil microbiome in rice fields suggests that microbial composition shifts in response to drought condition and the presence of plants. Among the bacteria involved, the phylum *Proteobacteria* appears to play the most important role in the survival of rice under drought condition.

## Findings

There is much research aiming to improve rice yields in order to feed a rapidly growing human population and improve its tolerance to climate change. In particular, drought stress, which is associated with global warming and desertification, greatly reduces rice production (Peng et al. 2004). Considerable progress has been made in identifying traits that promote drought tolerance in rice. However, it has not yet been possible to actually apply this knowledge due to the tradeoff between drought resistance and yield.

Advances in next generation sequencing (NGS) technology has made it possible to rapidly collect large amounts of genetic information. Among the various sequencing techniques, the development of amplicon sequencing of the 16S rRNA gene in prokaryotes has led to the field of metagenome analysis. Even before the advent of NGS sequencing techniques, interactions between plant and root microorganisms have been studied (Lethbridge and Davidson 1983; Germida et al. 1998; Brencic and Winans 2005). However, the use of amplicon sequencing methods has accelerated analysis of this interaction.

Diverse microbes interact with plants (Lugtenberg and Kamilova 2009; Berg et al. 2014). Thus, the power of amplicon sequencing analysis in rice has been applied to better understand the relationship between plants and microbes in the underground areas, namely, the soil, rhizosphere, rhizoplane, and endosphere (Arjun and Harikrishnan 2011; Sessitsch et al. 2012; Edwards et al. 2015). Moreover, microbiome analyses have proven useful in understanding the effects of external factors such as abiotic stress and fertilizer limitation (Ahn et al. 2012; Ikeda et al. 2014; Rothenberg et al., 2016). Amplicon sequencing has also been applied to rice under drought conditions (Santos-Medellin et al. 2017; Reim et al. 2017). However, amplicon sequencing data from different studies vary due to geographical differences and the presence or absence of plant. To better understand the relationship between the microbiome and drought condition, which are closely linked to rice productivity, we reanalyzed publicly available amplicon sequencing data on the soil microbiome of rice under drought condition. We identify a candidate phylum that plays a role in the plant’s response to the limits imposed by drought conditions on rice growth.

**Fig. 1:**
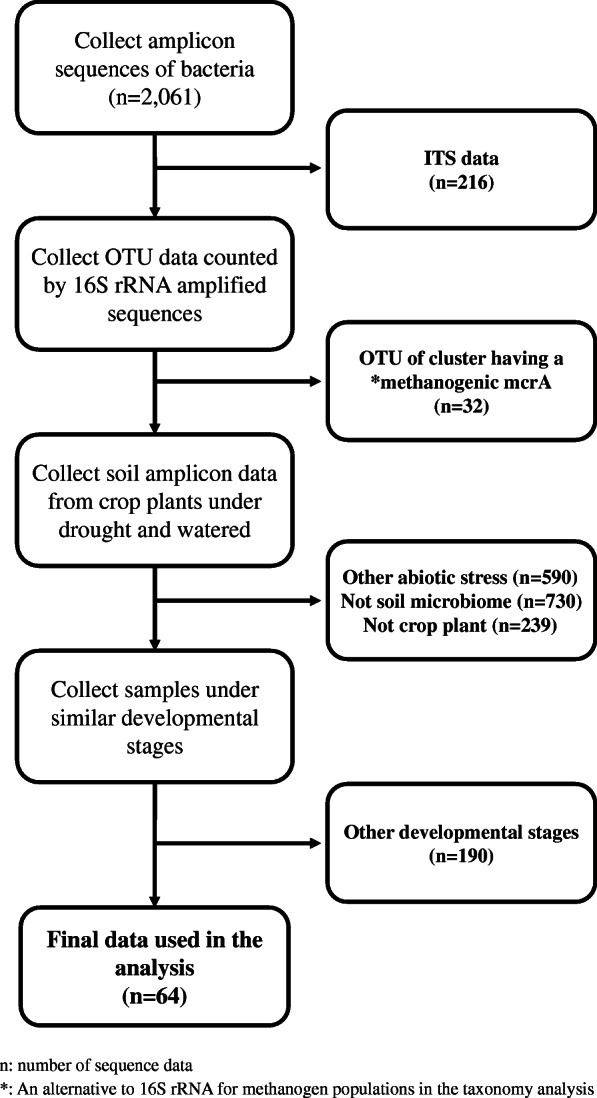
Workflow involved in data selection. In total, 2061 16S amplicon sequence datasets were collected from the National Center for Biotechnology Information (NCBI, https://www.ncbi.nlm.nih.gov/) and the European Molecular Biology Laboratory–The European Nucleotide Archive (EMBL-ENA, https://www.ebi.ac.uk/ena). Internal Transcribed Spacer (ITS) and methanogenic mcrA data were first removed. Then, abiotic stress data other than drought stress-treated root microbiome data and non-crop plant data were excluded. Finally, by removing data sampled at different developmental stages, we reanalyzed amplicon sequence data of crop plants under drought stress and at similar developmental stages

We collected 2061 microbiome sequence datasets from different plant species, sorted the data according to the workflow, and reanalyzed 64 soil microbiome sequences of rice (Fig. 1, Table S1). The microbiome data consists of data from soil, endosphere, rhizosphere, and rhizoplane (Table S1); however, the amount of microbiome data from the endosphere, rhizosphere, and rhizoplane is insufficient to allow rigorous comparison and re-analysis. Thus, we utilized only soil microbiome data for our analysis. We compared four different normalization methods: minmax, median, average, and total count, all of which showed similar patterns (Tables S2, S3, S4, and S5). Using normalized data from each sample, we selected the top 16 most abundant phyla (Table 1).

**Table 1:** Detailed information for the samples used to make Fig. 2 and Fig. 3

Alias	Specific name	Treatment^a^	Plant^b^	Source project
1	Watered treated soil from Arbuckle (U.S)	WATERED	O	PRJNA386367
2	Watered treated soil from Biggs (U.S)	WATERED	O	PRJNA386367
3	Watered treated soil from Davis (U.S)	WATERED	O	PRJNA386367
4	Drought treated soil from Arbuckle (U.S)	DROUGHT	O	PRJNA386367
5	Drought treated soil from Bigs (U.S)	DROUGHT	O	PRJNA386367
6	Drought treated soil from Davis (U.S)	DROUGHT	O	PRJNA386367
7	Watered treated irrigated soil (Thailand)	WATERED	X	PRJNA362531
8	Watered treated rainfed soil (Thailand)	WATERED	X	PRJNA362531
9	Drought treated irrigated soil (Thailand)	DROUGHT	X	PRJNA362531
10	Drought treated rainfed soil (Thailand)	DROUGHT	X	PRJNA362531

All replicates in each sample are averaged. In case of alias 7 and alias 8, incubated condition data is used out of fresh, re-incubated, drought, and recovery

^a^WATERED: well watered state; DROUGHT: water is drained from soil and dried state

^b^O: plant exists; X: plant does not exist

**Fig. 2:**
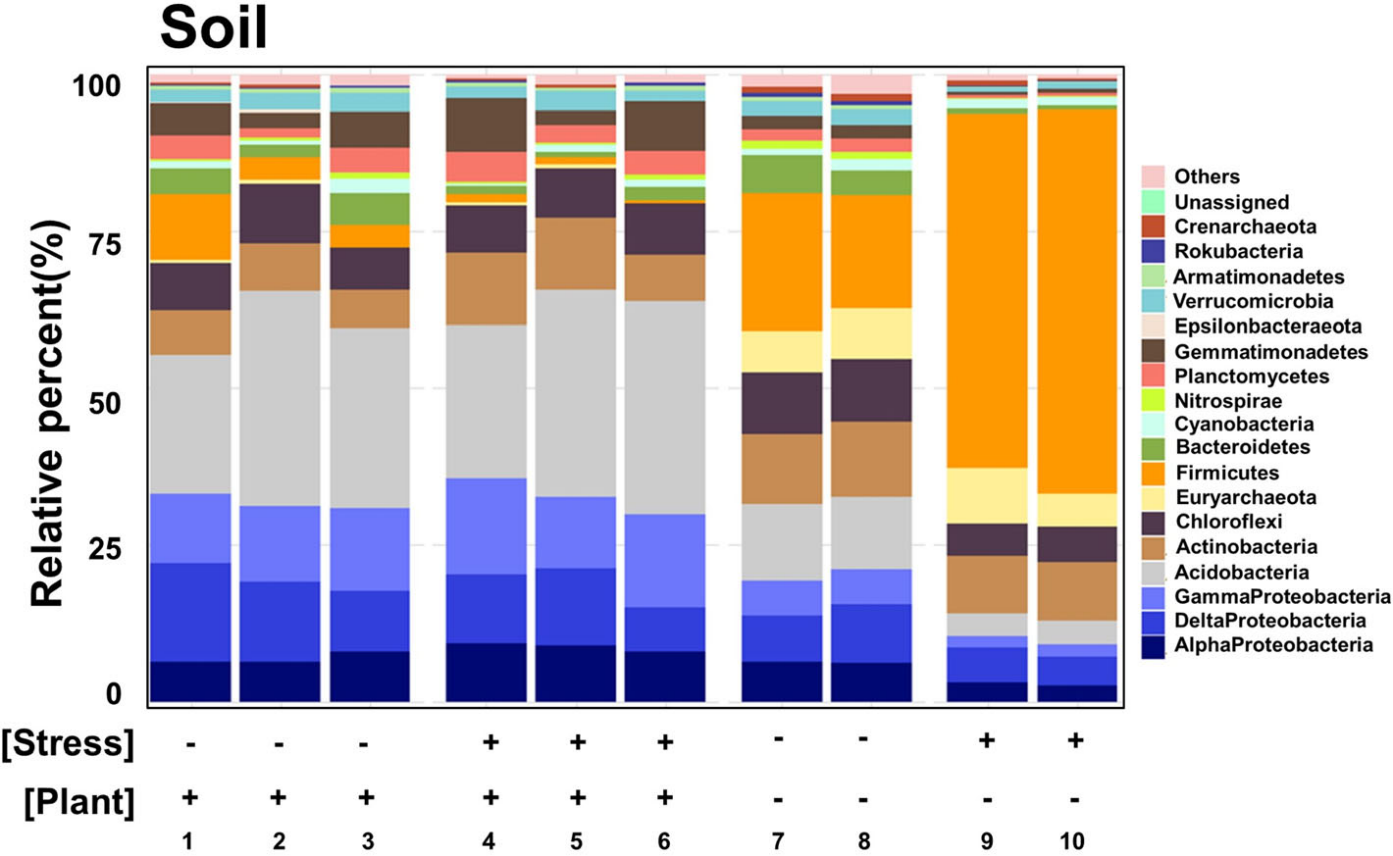
Relative abundance of the 16 most abundant phyla from each soil sample collected from systems under drought stress or the presence of plants. The data points represent the abundance of each phylum relative to the total of all phyla in each sample. The 16 most abundant phyla in each treatment are integrated and identified, as well as an unassigned phylum. Only *Proteobacteria* is subdivided into classes. Clusters of other phyla are labeled as “Others.” Grids are set according to the application of stress (drought (+)/watered (−)) and the presence of plants (with (+)/without (−)). Detailed information on each sample is presented in Table 1

Analysis of a stacked bar plot and alpha diversity boxplot (Fig. 2, and Figure S1) indicates that the predominant members of the microbiome under drought conditions changed according to the presence or absence of plants. A two-way ANOVA test confirms the significance of this microbiome shift in response to plants (Table S6). In addition, we compared the relative abundance levels between members of the microbiome community in different environmental conditions (Table S7). Based on the shift pattern of phyla, this analysis yielded the following four clusters: phyla affected by both factors, those not affected by both factors, those affected by only the presence of plants, and those affected by only drought condition (Table S6). Among these clusters, *Cyanobacteria* and *Epsilonbacteraeota* were not affected by either factor. Meanwhile, *Acidobacteria*, *Actinobacteria*, *Euryarchaeota*, *Gemmatimonadetes*, and *Planctomycetes* were affected by only one factor, i.e., the presence of plants. To focus only on soil microbiomes affected by drought conditions, we analyzed the two clusters formed with drought: that affected only by drought and that affected by the presence of plants and drought. To facilitate the comparison between the results of analysis in each condition, we calculated the relative abundance ratio of drought to watered condition (from here “ratio”).

Populations associated with drought conditions regardless of the presence of plants include *Chloroflexi, Nitrospirae, Rokubacteria*, and *Bacteroidetes.* In terms of the ratio of the abundance under drought to that under watered conditions, *Chloroflexi* shows a value of 0.738. In case of *Nitrospirae* and *Rokubacteria*, the ratios are 0.389 and 0.455, respectively (Table S8). Lastly, *Bacteroidetes* shows the lowest ratio of 0.250 (Table S8). Taken together, the microbiome developing under stress conditions is characterized by statistically significant shifts in the population that do not involve the establishment of a phylum with a relative abundance ratio greater than 1. This indicates that population of all phyla described in this section decreased under drought conditions (Table S6, Table S8, Figure S2). Therefore, we did not further explore this cluster to identify useful candidate phyla to assist rice in overcoming drought stress.

A recent study reporting on *Nitrospirae* focused mainly on the nitrogen cycle (Xue et al. 2016); knowledge about its relationship with drought is limited. Similarly, drought-related information on *Rokubacteria* is limited, especially since this phylum has been newly classified (Becraft et al. 2017). *Bacteroidetes* make up the largest portion of the microbiome of mammalian intestines, thus its characteristics have been revealed through human microbiome studies. However, the interactions between plants and *Bacteroidetes* is largely unknown. Literature searches on all the phyla in this cluster provided no clues on their functional significance in relation to drought. Thus, our search for candidate phyla that can help plants overcome drought condition focused on the cluster consisting of populations that are affected by the presence of plants and stress.

The populations affected by both drought conditions and the presence of plants are *Armatimonadetes*, *Verrucomicrobia*, *Firmicutes*, and *Proteobacteria*. The *Armatimonadetes* ratios of drought to watered are 0.819 in the presence of plants and 0.219 in the absence of plant (Table S7). The reduction in the relative abundance of *Armatimonadetes* is consistent with previous results in potato (Gumiere et al. 2019). Similarly, the *Verrucomicrobia* ratios of drought to watered are 0.896 in the presence of plants and 0.378 in the absence of plants (Table S7). In the presence of plants, the abundance levels of populations of *Armatimonadetes* and *Verrucomicrobia* were lower under drought conditions than those under watered conditions. However, their population levels increased when they were interacting with plants under drought conditions, compared to their levels without this interaction. Therefore, these phyla have potential significance in assisting plants to overcome drought stress.

In the case of *Firmicutes*, the ratios of drought to watered are 0.161 in the presence of plants and 2.935 in the absence of plants (Table S7). This result that shows the ratio increasing under drought conditions in the absence of plants and is consistent with previous findings that members of the *Firmicutes* community increase under drought conditions (Chodak et al. 2015). In the case of *Proteobacteria*, the ratios of drought to watered are 1.029 in the presence of plants and 0.484 in the absence of plants. Considering that the *p*-value of ANOVA-test is less than 0.05 and the ratio is greater than 1 in the presence of plants, *Proteobacteria* is selected as the phylum that is most affected by drought conditions in the presence of plants (Table S6, Table S7). In addition, *Proteobacteria* are noticeably reduced under drought conditions in the absence of plants, an observation that is consistent with previous research showing that the abundance of *Proteobacteria* is very low ratio in water-limited desert soil (Bu et al. 2018) (Table S7). Although we did not find any reports indicating that *Proteobacteria* populations may shift in response to drought conditions in the presence of plants, we nevertheless selected *Proteobacteria* as the best candidate phylum to assist plants in overcoming drought stress. Thus, we performed more detailed analysis on this phylum.

**Fig. 3:**
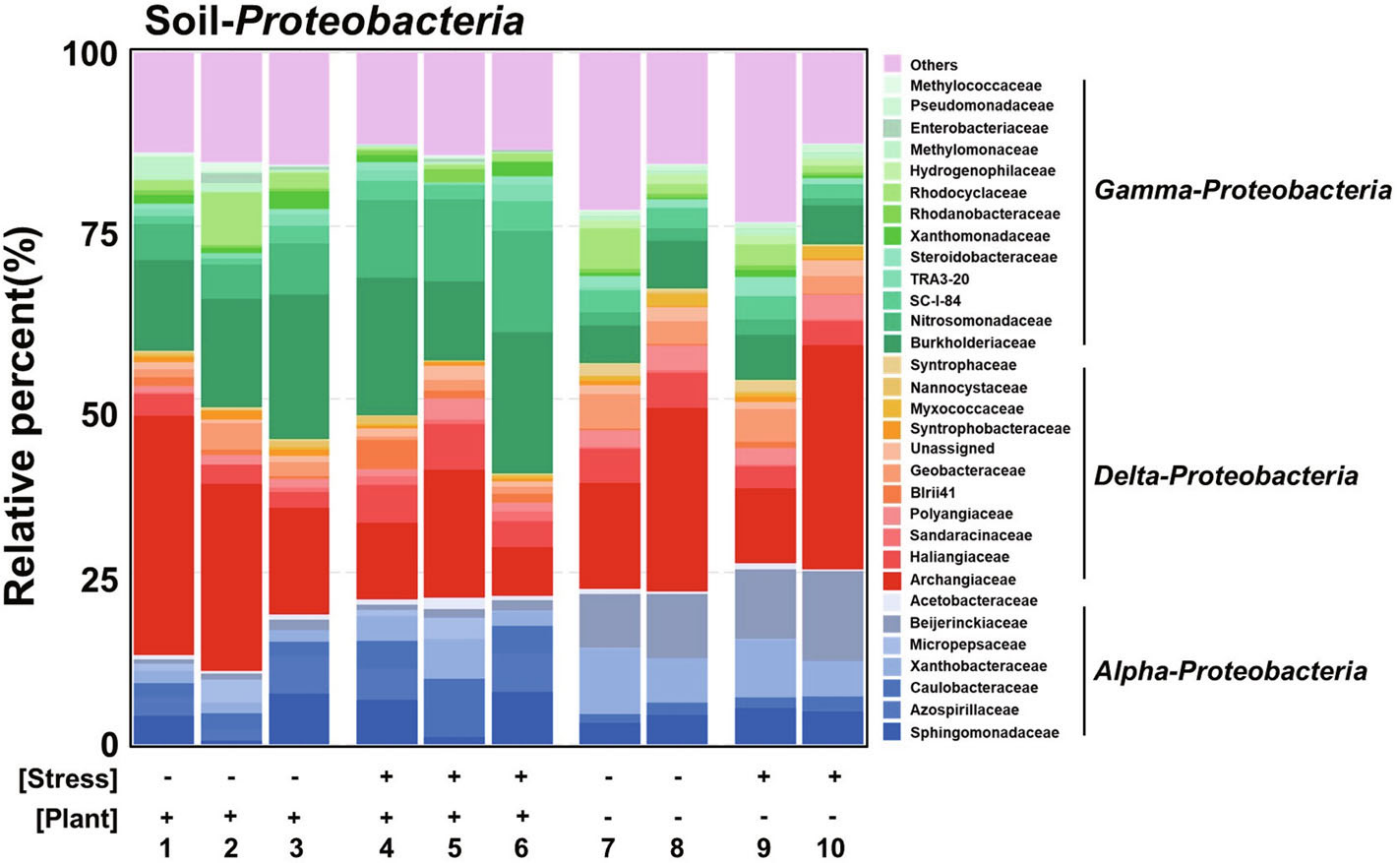
Relative abundance of the 30 most abundant *Proteobacteria* families from soil samples differentiated according to presence of drought stress or plants. Data points represent the abundance of each family relative to the total abundance of all families in each sample. The 30 most abundant families in each treatment were integrated, resulting in the identification of 31 families (one group consists of unassigned families). Each color represents a microbe in the following classes: *Alphaproteobacteria* (blue); *Deltaproteobacteria* (red); and *Gammaproteobacteria* (green). Bacteria not belonging to these classes are clustered as “Others.” Grids are set according to the application of stress (drought (+)/watered (−)) and presence of plant (with (+)/without (−)). Detailed information of each sample is presented in Table 1

To identify specific populations within *Proteobacteria* that respond to the presence of plants and drought, we analyzed the microbiome at the family level. The outcome of the analysis is presented in Fig. 3. The plot integrates 30 of the most abundant families in each sample. The following 13 families show a statistically meaningful cluster shift (*P* < 0.05) in response to both drought and the presence of plants: *Xanthobacteraceae, Acetobacteraceae, Beijerinckiaceae, Geobacteraceae, Syntrophobacteraceae, Syntrophaceae, SC-I-84, Xanthomonadaceae, Methylomonaceae, Rhodocyclaceae, Methylococcaceae, Steroidobacteraceae,* and *Hydrogenophilaceae* (Table S9). Among these families, *Xanthobacteraceae*, *Acetobacteraceae*, *Beijerinckiaceae*, *SC-I-84*, *Steroidobacteraceae*, and *Hydrogenophilaceae* share similar trends as that of *Proteobacteria* (Table S10)*. Xanthobacteraceae* is known to interact with plants and is involved in carbon and nitrogen cycling (Oren 2014; Wang et al. 2016). Meanwhile, members of *Acetobacteraceae* include plant growth promoting bacteria and well-known nitrogen-fixing bacteria (Saravanan et al. 2008; Reis and Teixeira 2015). In summary, we suggest six families belonging to *Proteobacteria* as candidates that assist plants cope with drought conditions.

**Fig. 4:**
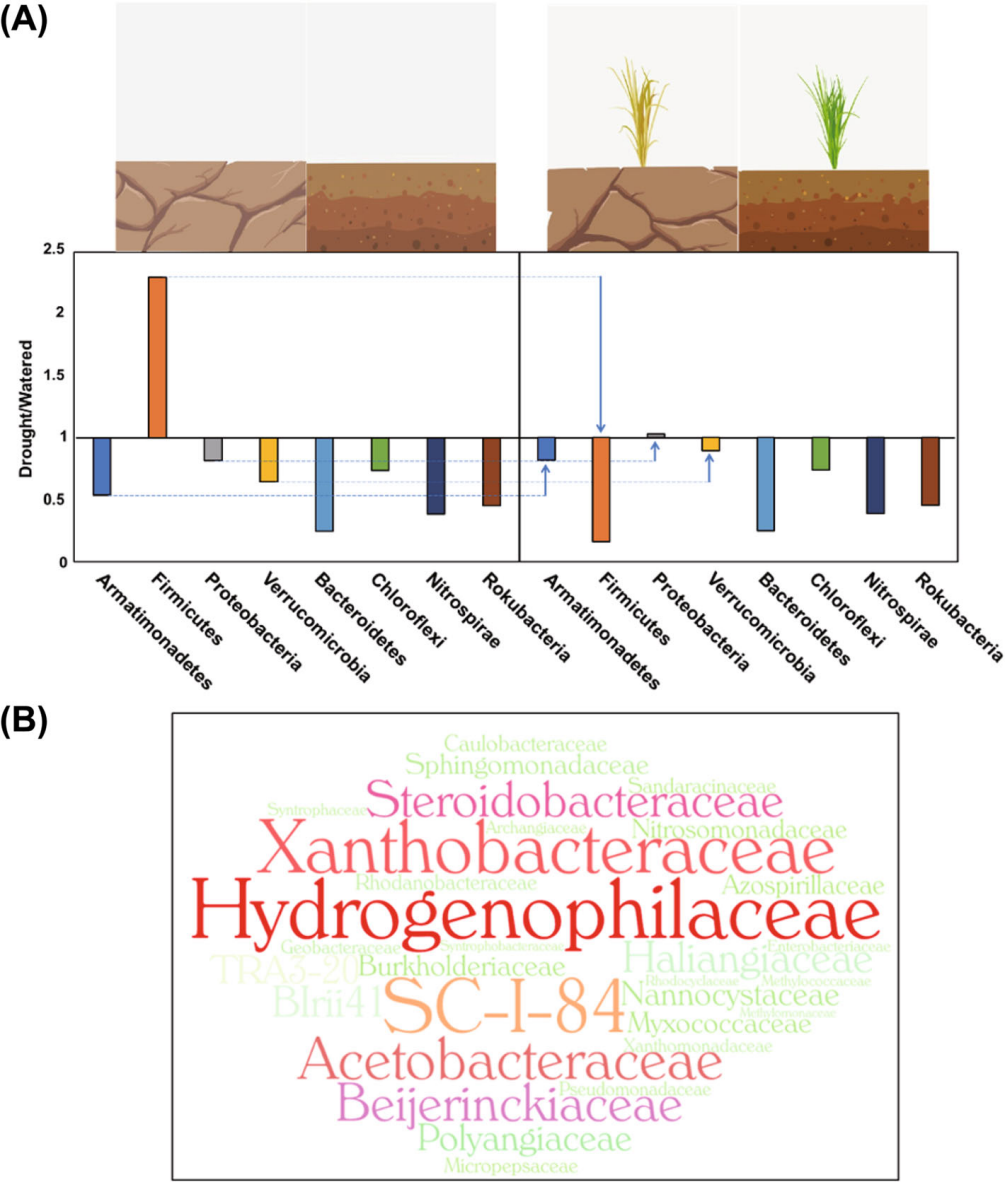
Model of the microbiome shift following a change in soil state and the presence of plants. The microbial shift produces two distinct clusters and suggests the dominance of six families belong to *Proteobacteria* under drought condition. **a** The left panel shows the ratio of drought to watered condition in the absence of plant for eight phyla and right one shows the ratio in the presence of plant for eight phyla selected from two clusters affected by drought. When watered soil with plants shift to drought with plants, phyla showing significant population shift are indicated with vertical arrows and dotted lines (*p*-value < 0.05; two-way ANOVA of both factors) (Table S6). The x-axis represents the ratio of drought to watered condition and y-axis indicates eight phyla significantly affected by drought. **b** The WordCloud refers to 13 families within the *Proteobacteria* that shift significantly as a cluster in the presence of both drought stress and plants (Table S9). Six families have statistically significant *p*-values and are presented in bigger size of letters with different color; these exhibit the same pattern as *Proteobacteria* in response to drought conditions in the presence of plants. The word sizes indicate the relative abundance ratio under drought conditions

Based on the results of our re-analysis, we constructed a model illustrating the statistically significant rice soil microbiome shift of four phyla in response to drought and the presence of plants; and four other phyla that are affected only by drought conditions. Figure 4 summarizes the abundance ratios of eight phyla affected by drought in the presence or absence of plants (Fig. 4a). Of these, *Armatimonadetes*, *Verrucomicrobia*, and *Proteobacteria* interacting with plants showed increased populations under drought conditions, while those of *Firmicutes* decreased. The *Proteobacteria*, in particular, includes more abundant populations under drought conditions than under watered conditions in the presence of plants, and was thus selected for the further analysis to identify six families that we propose as candidates that are worth further study (Fig. 4b). To help researchers in this field, we have analyzed the taxonomy of amplicons from reference studies that were excluded from our study due to insufficient amount of data (Figures S3, S4, and Table S11).

To compare the results of our analysis to those of other crops, we analyzed sorghum *(Sorghum bicolor L.)* data under drought (Figure S5). In this case, *Proteobacteria* did not display statistically significant population shifts. Thus, while additional experiments with *Proteobacteria* are recommended in rice, we cannot extend this recommendation to sorghum.

In conclusion, re-analysis of amplicon sequence data identifies several microbial shifts under drought conditions that depend on the presence of plants. We propose *Proteobacteria* as a suitable target for studies aiming to promote crop growth, in the same manner that previous studies have shown that inoculation of *Actinobacteria* enhances plant growth (Yandigeri et al. 2012; Hamedi and Mohammadipanah 2015). Because the composition of the microbiome depends largely on complex environmental variables and the genotype of the plant, new perspectives will be discovered as new microbiome data are generated.

## Supplementary information

**Additional file 1:.** Materials and Methods.

**Additional file 2: Figure S1.** Box plots of indexes for alpha diversity by drought condition (A) or by both factors (B) (Chao1, Faith_PD, Shannon, Simpson). Each index is calculated using the QIIME2 functions: *qiime diversity alpha-phylogenetic* and *qiime diversity alpha*.

**Additional file 3: Figure S2.** Bar plot of the integrated abundance of the 16 most dominant phyla in each environment. Each bar represents the mean value under each condition. Phyla that are not included in the integrated set of 16 phyla are classified as “others.”

**Additional file 4: Figure S3.** Bar plot of the integrated abundance of the 16 most dominant phyla from endosphere data collected under drought stress. Each bar represents the mean value under each condition. The 16 most abundant phyla in each treatment are integrated, resulting in the identification of 16 phyla, including unassigned phyla. Only groups within the *Proteobacteria* phylum are presented as classes. Phyla other than those classified are clustered as “Others.” Detailed information on each sample is presented in Table S11.

**Additional file 5: Figure S4.** Bar plot of the 16 most abundant phyla from rhizosphere and rhizoplane data collected under drought stress. Each bar represents the mean value under each condition. The 16 most abundant phyla in each treatment are integrated, resulting in the identification of 16 phyla, including unassigned phyla. Only groups within the *Proteobacteria* are presented as classes. Phyla other than those classified are clustered as “Others.” Detailed information on each sample is presented in Table S11.

**Additional file 6: Figure S5.** Bar plot of the integrated abundance the 16 most abundant phyla from sorghum under drought conditions. Ctrl, xxx, and DR refer to Control, well-watered, and drought state, respectively. Samples were grown under well-watered condition for 4 weeks to establish roots and then water was drained at 5 weeks after planting. Drought-stressed plants were sampled every week starting from 5 weeks after planting. Control samples were collected from a well-watered bathtub.

**Additional file 7: Table S1.** All amplicon sequence data collected for re-analysis.

**Additional file 8: Table S2.** MinMax of samples used in the analysis.

**Additional file 9: Table S3.** Median of the samples used in the analysis.

**Additional file 10: Table S4.** Average of the samples used in the analysis.

**Additional file 11: Table S5.** Total count of samples used in the analysis.

**Additional file 12: Table S6.***P*-value resulting from a two-way ANOVA accounting for drought state and presence of plant.

**Additional file 13: Table S7**. Relative abundance of the 16 most abundant phyla from each sample collected under drought in the presence of plants.

**Additional file 14: Table S8.** Relative abundance of the 16 most abundant phyla for each environmental condition.

**Additional file 15: Table S9.***P*-value resulting in identification of families of *Proteobacteria* from a two-way ANOVA according to drought state and presence of plant.

**Additional file 16: Table S10.** Relative abundance of the 30 most abundant families of *Proteobacteria* from each sample according to drought state and plant presence.

**Additional file 17: Table S11.** Details regarding sample data used for Figures S3 and S4.

## Data Availability

The datasets generated or analyzed in the current study are included in this article and its additional files. Detailed information about the all sequence data is mentioned in Table S1. All of OTU data we reanalyzed are prepared at https://github.com/WOOJONGHONG/Soil-Metagenome-reanalysis.

## Abbreviations

ANOVA: Analysis of Variance
NGS: Next Generation Sequencing
OTU: Operational Taxonomic Unit
