# Supplementary material for: Re-Analysis of 16S Amplicon Sequencing Data Reveals Soil Microbial Population Shifts in Rice Fields under Drought Condition
Source: Rice (N Y). 2020 Jul 2;13:44. doi: 10.1186/s12284-020-00403-6 (PMC7332601; doi:10.1186/s12284-020-00403-6)
Supplement: Supplementary file 1 — Additional file 1:. Materials and Methods. [file 12284_2020_403_MOESM1_ESM.docx]

**Dataset acquisition**

In total, 2,061 samples from 12 studies were reanalyzed, and the results for 64 sequences were included in our analysis. All of the data used for the metagenome analysis were downloaded from the Short Read Archive (SRA; http://www.ncbi.nlm.nih.gov/sra), NCBI PubMed (http://www.ncbi.nlm.nih.gov/pubmed), Google Scholar (http://www.scholar.google.com), and the European Nucleotide Archive (ENA; http://www.ebi.ac.uk/ena). In order to ensure data selection standardization, all data included samples from rice (Rhyzosphere and Endosphere) or paddy soil. Alternatively, data was gathered from samples that were undergoing abiotic stress conditions, but these samples were required to have a standard as a control.

**Preprocess of publicly available 16s rRNA amplicon-seq data**

All of the publicly available 16S rRNA amplicon-seq data was downloaded from the NCBI and the ENA and was processed through the QIIME2 software package (v. 2018.6.0), Python (v. 3.5.5), R (v. 3.5.1), and R studio (v. 1.1.442). Since all data had been already demultiplexed, no further demultiplexing analysis was performed. The quality of each sequence was trimmed at 20–30. For paired-end sequencing, *qiime vsearch join-pairs* was used to join the sequences. For paired-end sequences that were already joined, we set the sequence as *JoinedSequencesWithQuality* during data import. Further analysis proceeded following the QIIME2 workflow.

**Microbiome data analysis**

After importing each sample into a qza file, we used DADA2 and the denoise procedure to remove noise. Each individual classifier was trained with the primer used in each study and the read length from quality trimming. If there was no available information about the primer used, a pre-trained full-length SILVA classifier was used.

SILVA 132 QIIME release data was used as a reference data set. Data with 97% similarity was used for training classifiers based off of representative sequences and taxonomy analysis. OTU clustering proceeded with a closed reference approach, and reads with lower than 97% similarity were not used. Mitochondria and chloroplast taxonomy analysis were filtered through the *qiime taxa filter-table*.

**Statistical analysis using R**

Averages of taxonomy counts, plotted as a single value, were used if the data used in stacked bar plots had replicates. Each individual species in every sample was used as a ratio compared to the total OTU of each sample. The most abundant 10 bacteria in each sample were selected and integrated as a whole set. The others were clustered as “Others.” Only Proteobacteria was segmented into class levels for detailed analysis, as shown in Figure 2.

Only Proteobacteria was analyzed to the family level, as presented in Figure 3. The 10 most abundant species were selected and integrated as a whole set. The other species were clustered and labeled as “Others.” The analyzed data was classified and visualized depending on the location of the sample source, including soil, endosphere, and rhizosphere/rhizoplane. Only data of samples derived from soil were utilized in this paper. Data was grouped based on the presence or absence of a plant or drought stress: drought with plant, drought without plant, watered with plant, and watered without plant. R was used to calculate two-way ANOVA to compare treatments (drought or watered) and presence of a plant (with or without).

**Tools for visualization**

R (v. 3.5.1) and R studio (v. 1.1.442) were used to create all figures. Packages for drawing stacked bar plots and dot plots were ggplot2 (v. 3.5.1), ggthemes (v. 3.5.1), extrafont (v. 3.5.0), plyr (v. 1.8.4), scales (v. 1.0.0), and RColorBrewer (v. 1.1-2).
