# Supplementary figures and images for: Re-Analysis of 16S Amplicon Sequencing Data Reveals Soil Microbial Population Shifts in Rice Fields under Drought Condition
Source: Rice (N Y). 2020 Jul 2;13:44. doi: 10.1186/s12284-020-00403-6 (PMC7332601; doi:10.1186/s12284-020-00403-6)

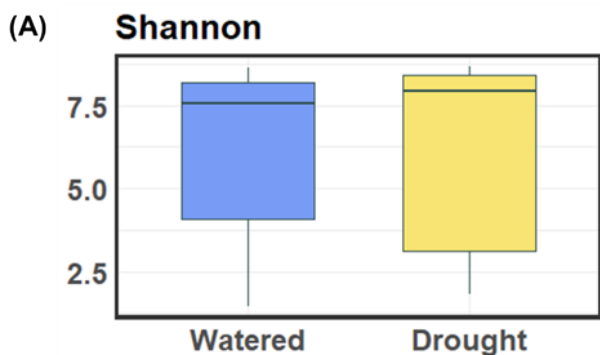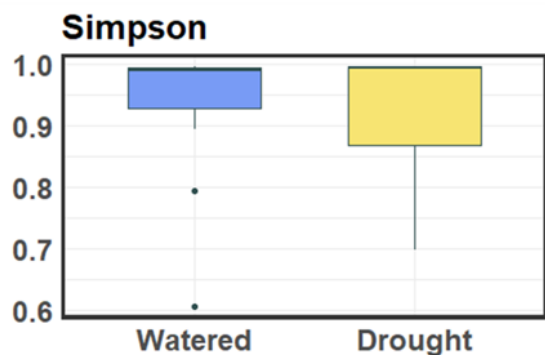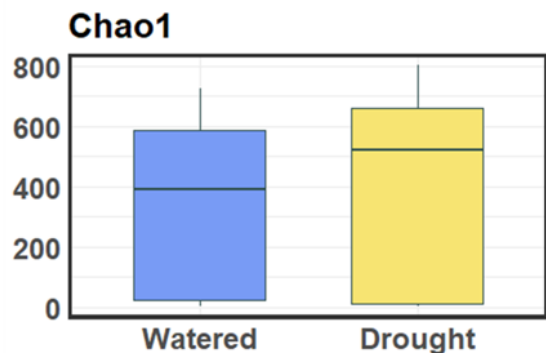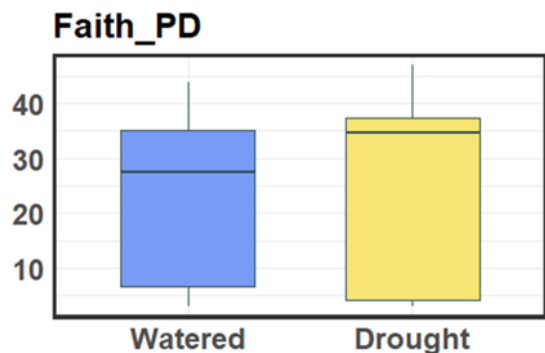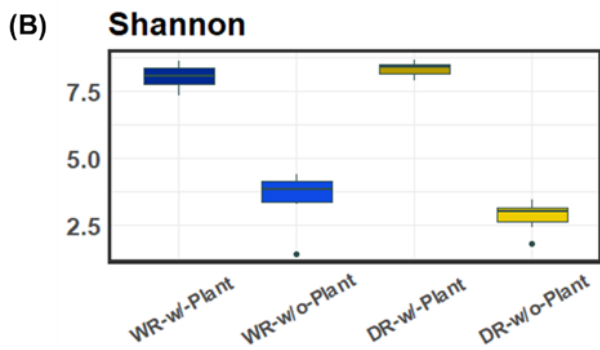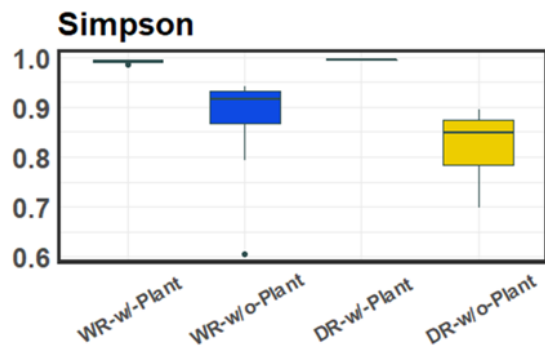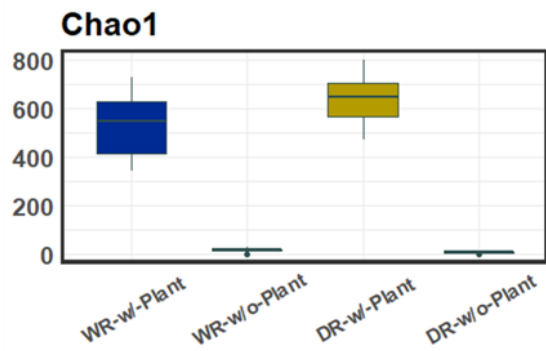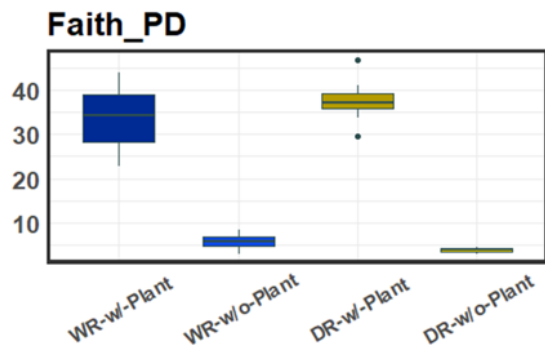

Supplement: Supplementary file 2 — Additional file 2: Figure S1. Box plots of indexes for alpha diversity by drought condition (A) or by both factors (B) (Chao1, Faith_PD, Shannon, Simpson). Each index is calculated using the QIIME2 functions: qiime diversity alpha-phylogenetic and qiime diversity alpha. [file 12284_2020_403_MOESM2_ESM.pdf]

# Soil

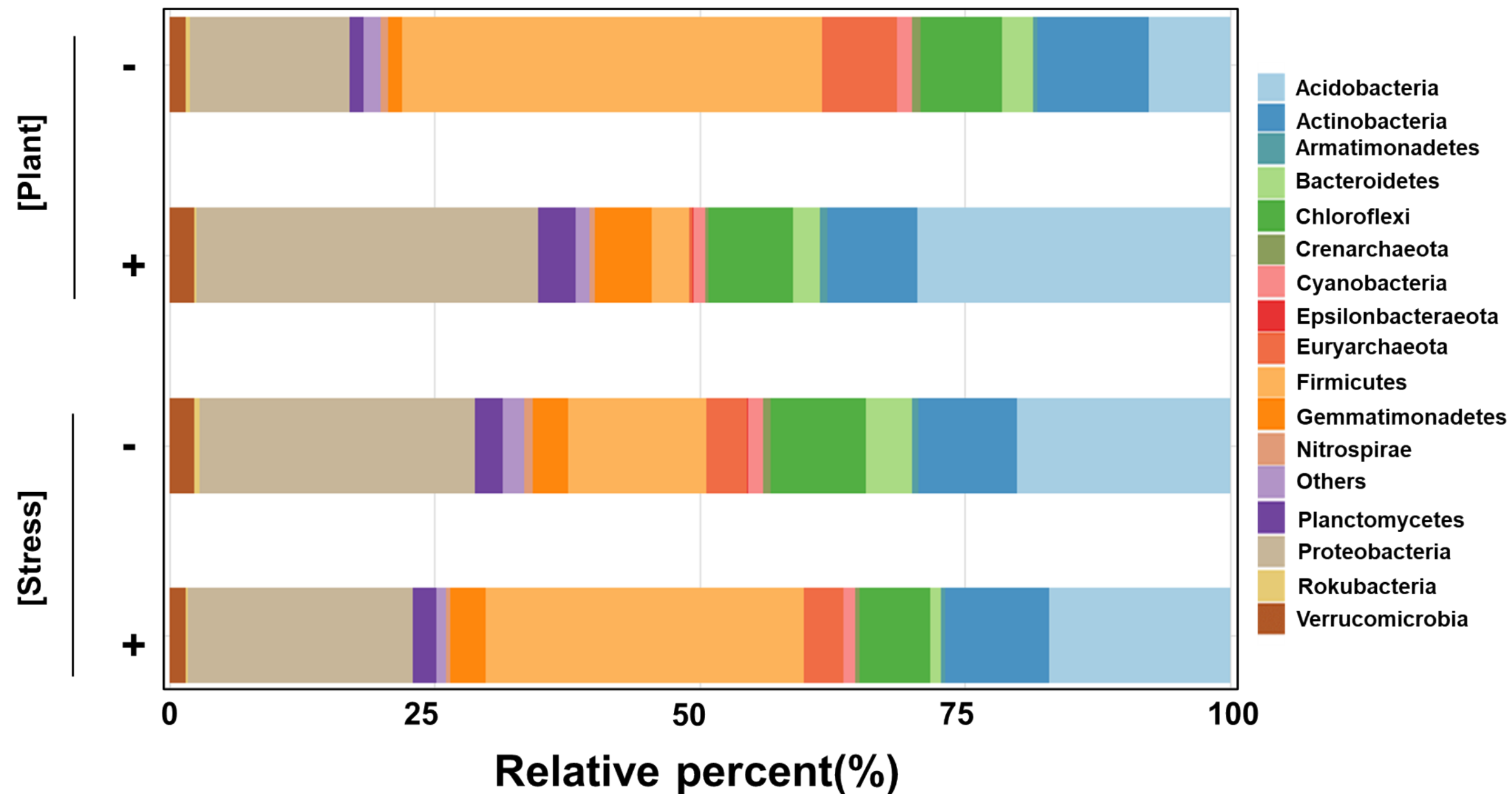

Supplement: Supplementary file 3 — Additional file 3: Figure S2. Bar plot of the integrated abundance of the 16 most dominant phyla in each environment. Each bar represents the mean value under each condition. Phyla that are not included in the integrated set of 16 phyla are classified as “others.” [file 12284_2020_403_MOESM3_ESM.pdf]

# Endosphere

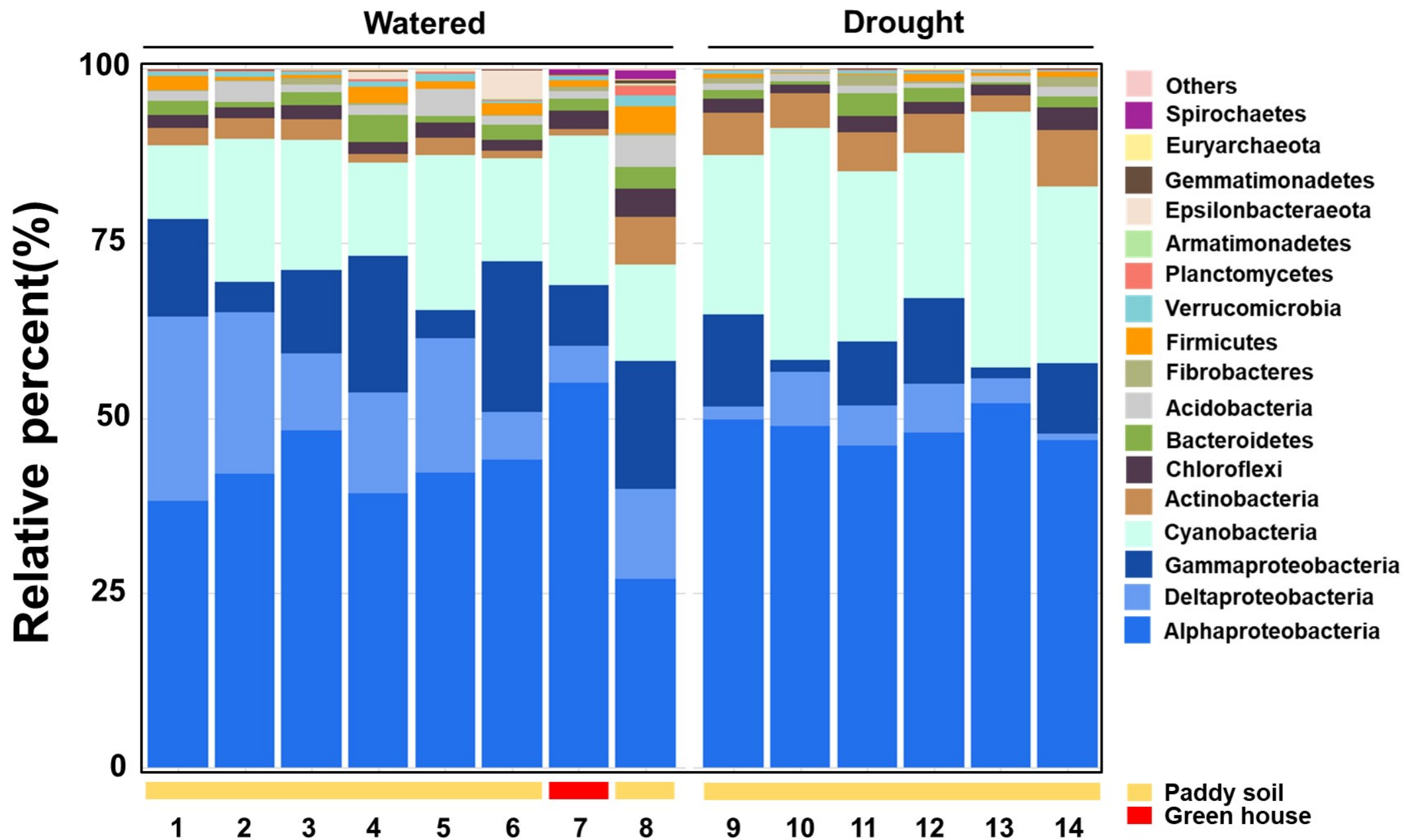

Supplement: Supplementary file 4 — Additional file 4: Figure S3. Bar plot of the integrated abundance of the 16 most dominant phyla from endosphere data collected under drought stress. Each bar represents the mean value under each condition. The 16 most abundant phyla in each treatment are integrated, resulting in the identification of 16 phyla, including unassigned phyla. Only groups within the Proteobacteria phylum are presented as classes. Phyla other than those classified are clustered as “Others.” Detailed information on each sample is presented in Table S11. [file 12284_2020_403_MOESM4_ESM.pdf]

# Rhizosphere/Rhizoplane

Watered

Drought

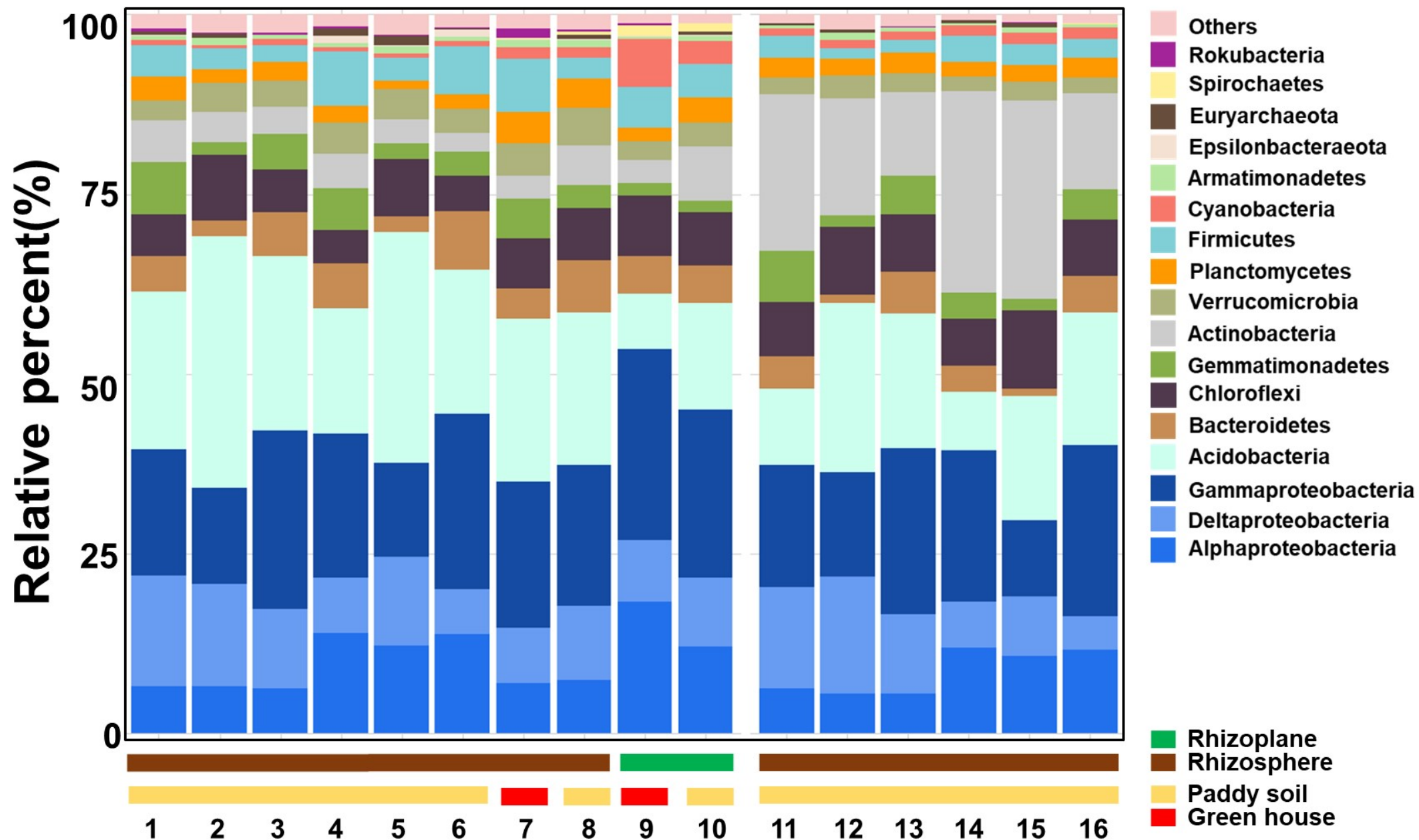

Supplement: Supplementary file 5 — Additional file 5: Figure S4. Bar plot of the 16 most abundant phyla from rhizosphere and rhizoplane data collected under drought stress. Each bar represents the mean value under each condition. The 16 most abundant phyla in each treatment are integrated, resulting in the identification of 16 phyla, including unassigned phyla. Only groups within the Proteobacteria are presented as classes. Phyla other than those classified are clustered as “Others.” Detailed information on each sample is presented in Table S11. [file 12284_2020_403_MOESM5_ESM.pdf]

# Soil

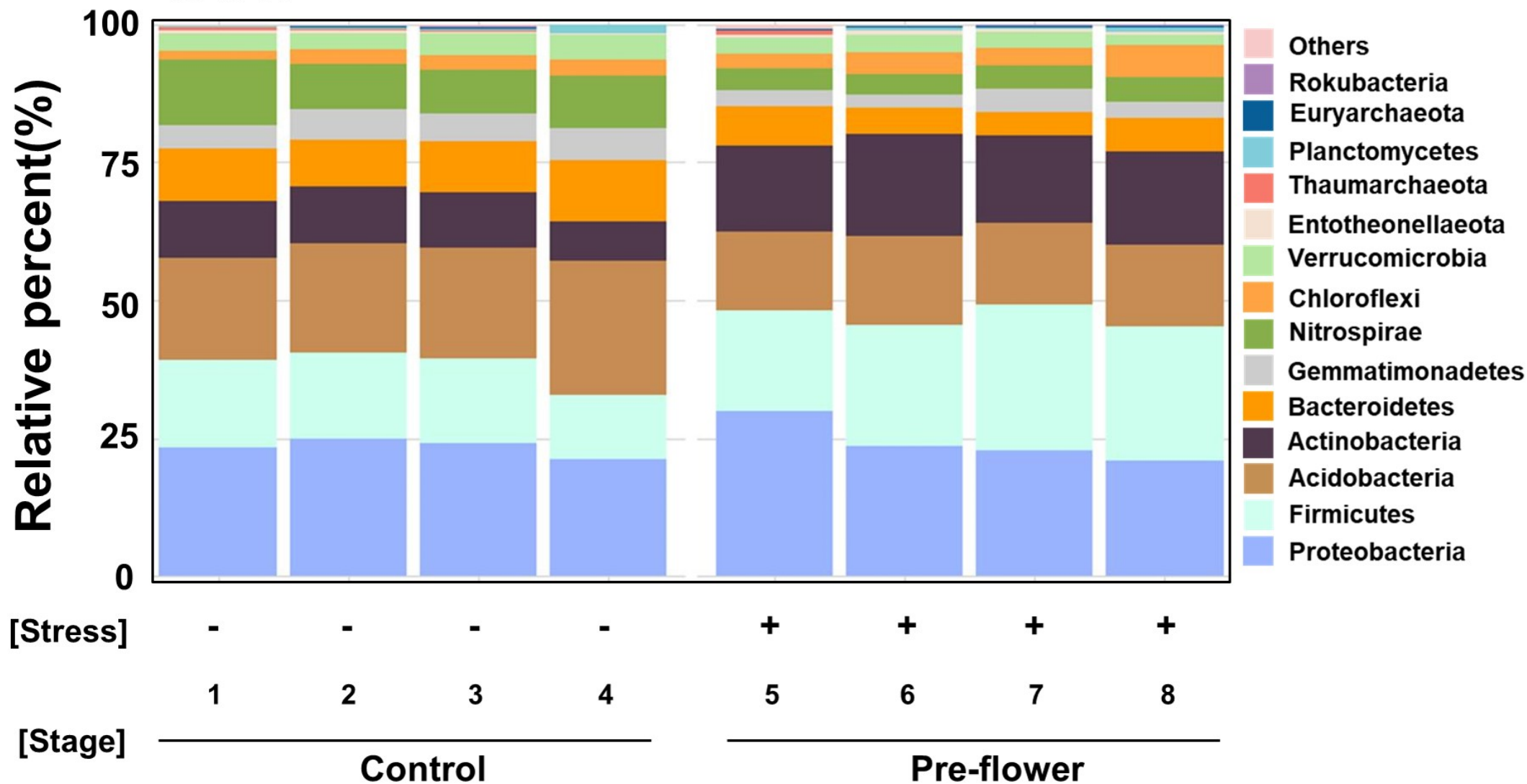

Supplement: Supplementary file 6 — Additional file 6: Figure S5. Bar plot of the integrated abundance the 16 most abundant phyla from sorghum under drought conditions. Ctrl, xxx, and DR refer to Control, well-watered, and drought state, respectively. Samples were grown under well-watered condition for 4 weeks to establish roots and then water was drained at 5 weeks after planting. Drought-stressed plants were sampled every week starting from 5 weeks after planting. Control samples were collected from a well-watered bathtub. [file 12284_2020_403_MOESM6_ESM.pdf]
