# Supplementary material for: Re-Analysis of 16S Amplicon Sequencing Data Reveals Soil Microbial Population Shifts in Rice Fields under Drought Condition
Source: Rice (N Y). 2020 Jul 2;13:44. doi: 10.1186/s12284-020-00403-6 (PMC7332601; doi:10.1186/s12284-020-00403-6)
Supplement: Supplementary file 7 — Additional file 7: Table S1. All amplicon sequence data collected for re-analysis. [file 12284_2020_403_MOESM7_ESM.docx]

| No. of  Samples | Hypervariable  Region(s) | Avg sequencing depth per sample | Sequencing platform | Sample type(s) | Study observation(s) | Country of origin | Data availability (accession no.) |
| --- | --- | --- | --- | --- | --- | --- | --- |
| 497 | V4-V5 | 51,970^b^ | Illumina MiSeq | *Oryza Sativa* | To uncover factors driving differences in microbial diversity for root associated microbial communities in rice | USA | PRJNA255789 |
| 432 | V4 | 29,910^b^ | Illumina MiSeq | *Oryza Sativa Oryza glaberrima*  Greenhouse Soil | To analyze the drought-mediated compositional shifts in the microbial communities assembled in the rhizosphere and endosphere of a diverse set of rice accessions grown in different agricultural soils | USA | PRJNA386367 |
| 32  32 | V4 | 748,158^c^  692,396^c^ | Illumina MiSeq | Paddy Soil | To investigate structure and function of the methanogenic microbial communities are different in irrigated and rain-fed paddies and react differently upon desiccation stress | Thailand | PRJNA362531  PRJNA362529 |
| 5 | V4 | 78,479^b^ | Illumina MiSeq | Paddy Soil | To investigate the microbial community of paddy soil contaminated with Fe-S-rich acid mine drainage | China | PRJNA260992 |
| 45 | V3-V4 | 10,619^b^ | 454 GS FLX  Titanium | Paddy Soil | To explore the divergence of microbial community structure among iron plaque, bulk soil and rhizosphere soil of paddy field | China | PRJNA259434 |
| 1 | NA^a^ | 179,338^c^ | 454 GS FLX+ | Paddy Soil | To study the community structures after the addition of ammonium and nitrate | China | PRJNA248059 |
| 740 | V3-V4 | 40,863^b^ | Illumina MiSeq | *Sorghum bicolor* | To explore the relationship between drought and microbial recruitment in *Sorghum bicolor*. | USA | PRJNA435634 |
| 36 | V1-V3 | 2,265^b^ | 454 GS FLX  Titanium | Paddy Soil | To investigate the bacterial and archaeal diversity in the rice paddy soils which have been fertilized for 23 years | Korea | PRJNA169177 |
| 2 | V3-V4 | 558,950^c^ | Illumina HiSeq 1000 | *Oryza sativa* | To investigate the root endophytic microbial community present in the local cultivar of rice (*Oryza sativa L.*) at different field condition of West Bengal | India | PRJNA360379 |
| 5 | V1-V3 | 60,936^c^ | 454 GS FLX  Titanium | *Oryza sativa* | To investigate bacterial cell communities inhabiting roots of rice, which were grown in paddy fields under low N (LN; 0kg ha-1) and standard N (SN; 30kg ha-1) fertilization conditions in 2009. Prior to the experiments, LN fields has not been subjected to N fertilization for consecutive 5 years. | Japan | PRJDA61421 |
| 239 | V3-V4 | 18,458^c^ | Illumina MiSeq | Grassland | To investigate how plant communities moderate belowground response top drought. Treatments consisted of different dominant species and levels of evenness of four common grassland species, which were subjected to a drought | UK | PRJEB27398 |

**Table S1.** All amplicon sequence data collected for re-analysis

*^a^*NA, not available.

*^b^*Reads after chimera removal

*^c^*Reads without chimera removal
